# Schiff Bases From 4-Aminoantipyrine: Investigation of Their In Silico, Antimicrobial, and Anticancer Effects and Their Use in Glucose Biosensor Design

**DOI:** 10.1155/bca/2786064

**Published:** 2025-04-01

**Authors:** Aşkın Erbaş, Selinsu Dikim, Fatma Arslan, Onur Can Bodur, Seza Arslan, Fatma Özdemir, Nurşen Sarı

**Affiliations:** ^1^Graduate School of Natural and Applied Sciences, Gazi University, Ankara, Türkiye; ^2^Department of Chemistry, Faculty of Arts and Science, Kutahya Dumlupinar University, Kutahya, Türkiye; ^3^Department of Chemistry, Faculty of Science, Gazi University, Ankara, Türkiye; ^4^Department of Biology, Faculty of Arts and Sciences, Bolu Abant Izzet Baysal University, Bolu, Türkiye

**Keywords:** 4-aminoantipyrine, antimicrobial activity, biosensor, DNA cleavage, glucose, in silico

## Abstract

Five new Schiff bases from 4-aminoantipyrine were synthesized, characterized, and evaluated for their antimicrobial and DNA cleavage activities, and drug similarity properties and cytotoxicity prediction using in silico analysis. All Schiff bases had good antibacterial and antifungal activities. All compounds showed self-activating DNA cleavage ability in the absence of any reductant or oxidant at low concentrations. Modified carbon paste electrodes were prepared with all Schiff bases, and a glucose biosensor was designed. Schiff base coded (4AA-Fc) was found to have the best sensitivity to H_2_O_2_. It was observed that the prepared biosensor has a working range at low concentrations (1.0 × 10^−7^–1.0 × 10^−6^ M (*R*^2^ = 1.0)) and a low detection limit (1.0 × 10^−8^ M). At the same time, 4AA-Fc was found to be a potent compound for bactericidal and fungicidal effect, killing pathogens. Thus, it could be used for the development of a resistant biosensor in external environment. It also showed a complete DNA degradation. In silico ADME analysis and cell line cytotoxicity studies found these new Schiff bases to have favorable drug-like properties, indicating potential for the development of therapeutic drugs. In particular, the compounds were not a P-gp substrate. Thus, they could be a potential anticancer agent. The present study may be useful for further scientific research in the field of the design, synthesis, and biological studies of bioactive substances.

## 1. Introduction

4-aminoantipyrine is a heterocyclic molecule with two nitrogen atoms in its ring joined by a carbonyl functional group and a highly reactive amine. The existence of heteroatoms affects the electron redistribution and gives an aromatic character, known as the heteroatom effect [1], which confers reactivity, chelating action, and other properties. As a result, it is used in a variety of research areas such as analytical, modern organic, bioorganic, and medicinal chemistry [2–4].

**Figure 1:**
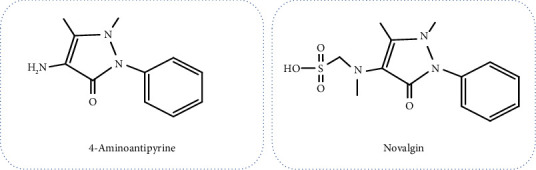
Structure of 4-aminoantipyrine and novalgin molecules.

4-aminoantipyrine (4-aminophenazone) was first synthesized in the late nineteenth century by Friedrich Stolz and Ludwig Knorr and sold as Pyramidon, an antipyretic drug [2, 5]. It is a metabolite of aminopyrine and metamizole. The prodrug metamizole with analgesic, antipyretic, and anti-inflammatory effects was first used medically in Germany under the brand of Novalgin (Figure 1). Nonenzymatic breakdown of the metamizole results in 4-N-methylaminoantipyrine (4-MAA), which is then N-demethylated to 4-aminoantipyrine (4-AAP), which enters the systemic circulation [6]. 4-AAP can form stable complexes with heme and has apparent denaturing effects on transportable proteins in the circulatory system [3, 7]. 4-AAP is an aromatic compound with analgesic, anti-inflammatory, and antipyretic properties [8, 9]. However, because of the risk of agranulocytosis, 4-AAP is rarely used as an analgesic nowadays [3].

Regarding biochemical reactions and environmental monitoring, 4-AAP is the most commonly used for the colorimetric determination of phenolic compounds in wastewater, pesticides, and fungicides in the presence of alkaline oxidizing agents. Furthermore, it is used as a reagent in a wide variety of analytical applications, such as glucose determination in the presence of phenol as well as peroxidase and uric acid determination using hydrogen peroxide [3, 4, 8].

Recently, 4-AAP and its Schiff base derivatives have attracted considerable attention for various application including analytical reagent, sensor development, catalysis, intermediates in organic synthesis, and new drug development [3, 4, 10, 11]. Schiff bases are aldehyde- or ketone-like compounds in which the carbonyl group is replaced by an imine (> C=N–) or azomethine (-HC=N-) exhibiting a broad range of biological activities such as antibacterial, antifungal, anticancer, antimalarial, anti-inflammatory, cytotoxicity, DNA binding, and DNA cleavage activities [4, 9, 10, 12–14].

Antimicrobial drugs are used to kill or prevent the growth of the microorganisms to cure infections in humans and animals. However, the intensive use or misuse of available drugs in the treatment of infections has led to the emergence of resistant strains of microorganisms [15, 16]. Therefore, researchers in the pharmaceutical industry are continuously focusing on finding highly promising drug candidates, but getting such compounds is not an easy process [4, 16, 17]. The antimicrobial activity of Schiff base derivatives of 4-AAP against various microorganisms was reported in previous studies [9–11, 18].

Cleavage of DNA affects many biological activities, including DNA replication, transcription, and recombination. In addition, the cleavage activity is an effective mechanism for the development of therapeutic agents, biosensor design, and gene editing [19]. Chemotherapy plays a crucial role in clinical treatment of cancer, and the scientists have researched new anticancer agents for the success treatment. It is known that the capability of DNA cleavage is an important property for the potential of DNA-targeted cancer agents [20]. Recently, studies involving the development of self-activating chemical nucleases as DNA therapeutic candidates without any exogenous redox agents have gained importance for the discovery of new chemotherapeutic drugs and properties that effect DNA structure and conformation [21]. Besides, in many studies, DNA cleavage activity has been also considered as a suitable property for finding new therapeutic agent candidates [22, 23].

*In silico* studies have become an increasingly valuable tool in recent years, providing an efficient and cost-effective method for the identification of drug candidates [24, 25], because predicting the pharmacokinetics and toxicity of target compounds provides information about their potential efficacy and safety, thereby accelerating the drug discovery process and improving success rates [24].

Since maintaining normal blood glucose levels is recommended, various glucose biosensors have been developed to provide accurate and reliable glucose monitoring [26]. Glucose biosensors are particularly in urgent demand in the medical field. Glucose biosensors today dominate the biosensor market [26, 27]. Glucose biosensors usually involve the immobilization of glucose dehydrogenase (GDH) or glucose oxidase (GOx) on electrodes modified with different materials; however, the use of GOx is often preferred because this enzyme shows higher affinity and sensitivity to glucose and remains stable over wider temperature and pH ranges [28].

Although 4-AAP has significant denaturing effects on heme and transportable proteins in the circulatory system [3, 4, 7, 29], some studies have focused on the enhancement of its favorable effects by Schiff base formation. In particular, there are few studies on preparing biosensors modified with Schiff bases with antibacterial properties [30]. As it is known, the ability of any material to be used to prevent viral or bacterial infections becomes more important in pandemic periods. The pharmaceutical industry has increased its attempts to develop new drugs, such as anticancer, antibacterial, and antifungal agents [16, 17]. Thus, the present study aimed at the synthesis, detection of antimicrobial and DNA cleavage activities, and glucose biosensor design of novel five Schiff bases derived from 4-aminoantipyrine. Furthermore, it was evaluated the in silico prediction of their absorption, distribution, metabolism, and excretion (ADME) and cytotoxicity properties in this study.

## 2. Experimental Section

### 2.1. Materials and Methods

The materials were all of reagent grade (Sigma-Aldrich Company). A Thermo Scientific Flash 2000 organic elemental analyzer device was used for the elemental studies. NMR spectra of the studied Schiff bases were recorded with a Bruker Spectrospin Avance DPX-400 instrument using TMS as internal standard and DMSO-d_6_ as solvent. IR spectra were recorded range from 4000 to 450 cm^−1^ on a Mattson-1000 FT-IR instrument. Spectroscopic DNA binding was determined using an ultraviolet–visible (UV–vis) spectrophotometer (Shimadzu-1800, ENG240V) in the range of 1100–200 nm.

Reference microorganisms: For antibacterial activity tests, Gram-positive bacteria used were *Listeria monocytogenes* RSKK 475, *Bacillus cereus* ATCC 14579, *Bacillus subtilis, Bacillus thuringiensis, Staphylococcus aureus* ATCC 6538, *Staphylococcus aureus* ATCC 25923, *Staphylococcus aureus* ATCC 29213, *Staphylococcus epidermidis* ATCC 12228, and S*treptococcus pyogenes* ATCC 19615. Gram-negative bacteria were *Escherichia coli* ATCC 8739, *Escherichia coli* ATCC 25922, *Enterobacter cloacae* NCIMB 13597, *Enterobacter cloacae* ATCC 23355, *Klebsiella pneumoniae* ATCC 700603, *Pseudomonas aeruginosa* ATCC 27853, *Salmonella* Typhimurium ATCC 13311, *Salmonella* Typhimurium ATCC 14028, and *Salmonella* Enteritidis ATCC 13076. Determining the antifungal activity, five yeast strains were used: *Candida albicans* ATCC 10231, *Candida albicans* ATCC 90028, *Candida albicans* ATCC 200955, *Candida parapsilosis* ATCC 22019, and *Candida krusei* ATCC 6258. Strains were stored as glycerol stocks −20°C before use. For DNA cleavage assay, pBR322 plasmid DNA (Thermo Scientific), agarose gels (Prona Agarose Biomax, European Economic Community), ethidium bromide (Sigma-Aldrich), and tris–borate–EDTA (TBE) buffer (Sigma-Aldrich) were used. Visualization of gels was performed using a UV transilluminator (DNR Minilumi Bio-imaging Systems Ltd., Jerusalem, Israel).

Equipment and reagents for glucose biosensor design:CHI 1230-B model electrochemical workstation was used in electrochemical experiments. A 0.3 cm diameter carbon paste electrode was used as working electrode, Ag/AgCI with BAS RE-5B as reference electrode, and platinum wire with MW-1032 as counter electrode. GOx (purified from *Aspergillus niger*, 10,000 units/mg) was obtained from Sigma. Glucose was obtained from Merck.

## 3. Synthesis of Schiff Bases and Their Antimicrobial and Anticancer Effects and Glucose Biosensor Design

### 3.1. General Synthesis of Schiff Base Including 4-Aminoantipyrine; (4AA-Fc), (4AA-SA), (4AA-FSA), (4AA-ClFSA), and (4AA-FMeSA)

4-aminoantipyrine (aminophenazone), which was first synthesized by Friedrich Stolz and Ludwig Knorr, was sold as an antipyretic drug [2, 5]. The aromatic substance containing three nitrogen and one carbonyl group with analgesic, antipyretic, and anti-inflammatory effects was first used in Germany under the brand name “Novalgin” (Figure 1).

In this study, a mixture of 4-aminoantipyrine 0.625 × 10^−3^ mol in EtOH (10 mL) was added dropwise to the 10 mL hot EtOH solution of 0.625 × 10^−3^ the aldehyde (ferrocenecarboxaldehyde, salicylaldehyde, 5-fluorosalicylaldehyde, 3-chloro-5-fluorosalicylaldehyde, 5-fluoro-3-methylsalicylaldehyde) and heated under reflux for 2 h at the boiling point of alcohol (78°C). Thin-layer chromatography (TLC) (hexane/ethyl acetate: 3/1) was used to monitor the progress and completion of the reaction for 2 h. Without cooling, the mixture was quickly filtered through sieve paper and left to stand. The mixture was collected by filtration after a further seven days at laboratory temperature (18°C), washed two more times with 10 mL C_2_H_5_OH, and then filtered through filter paper and dried at room temperature. The yield (%) of the products is given in Table 1.

**Figure 2:**
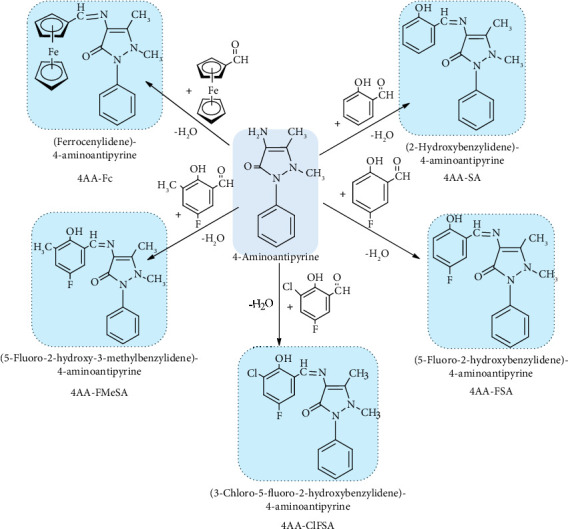
Structure of Schiff bases derived from 4-AAP.

Five newly synthesized Schiff bases derived from 4-AAP (4-aminoantipyrine) were 4AA-Fc, 4AA-SA, 4AA-FSA, 4AA-ClFSA, and 4AA-FMeSA, and their structures are given in Figure 2.

### 3.2. Experiments of Antimicrobial Activity

Antibacterial activity of the test compounds against bacterial strains was performed using the broth microdilution method according to CLSI guidelines [31]. All strains were grown in tryptic soy agar plates (Merck, Darmstadt, Germany) for 24 h at 37°C. After incubation, several colonies on the plates were suspended in Mueller–Hinton broth (MHB) (Merck, Darmstadt, Germany) to adjust the bacterial suspension turbidity to 0.5 McFarland standard (1 × 10^8^ colony-forming units per mL—CFU/mL). The adjusted suspension was subsequently diluted to a final concentration of 5 × 10^6^ CFU/mL. The serial dilutions of the test chemical stock solution prepared in DMSO (2000 μM) were ranged from 1000 to 1.95 μM using MHB in a 96-well microtiter plate (Lp Italiana, U-bottom), then an equal volume of the final bacterial inoculum was added to each well of the 96-well plate. The antimicrobial ampicillin (Sigma) was added to each plate as a positive control. After incubation at 37°C for 24 h, the plates were read with a microplate reader (Thermo Electron Corporation, Vantaa, Finland). Ampicillin was also used as a reference standard for antibacterial activity of test bacteria. The minimum inhibitory concentration (MIC) values were determined as the lowest concentration of the test chemical which inhibits the growth of bacterial strains. In addition, the minimum bactericidal concentration (MBC), defining as the lowest concentration of an antimicrobial necessary to kill a given organism after subculture onto antibiotic-free media, was determined. For that, after reading, 10 μL of the first six dilutions, ranging from 1000 to 31.25 μM, in the microtiter plate was seeded as a spot inoculation onto MHB (compound-free) and incubated (37°C for 24 h) to determine viable cells. The antifungal activity of the compounds was also evaluated against reference strains of the yeast *Candida* using CLSI standards [32]. Experiments for the MIC values were performed using the broth microdilution method in 96-well plates. Later, the minimum fungicidal concentration (MFC) of the test compounds against five yeast strains was determined. Briefly, the *Candida* strains were cultivated on potato dextrose agar (PDA) (Merck, Germany) as an antimicrobial growth-free medium and incubated at 35°C. After incubation, several colonies selected from the PDA plate were inoculated into sterile saline (0.85% NaCl) to adjust the cell density to the 0.5 McFarland standard (5 × 10^6^ cells per mL). This adjusted yeast stock solution was diluted with RPMI 1640 broth medium (Sigma-Aldrich R8758) to a final concentration of 5 × 10^3^ cells per mL. Twofold serial dilutions of the tested compounds in RPMI 1640 broth in the microtiter plate were prepared, and then the yeast suspension was added to each well. Thus, the concentration range of 1000 to 1.95 μM of the tested compounds was applied. Fluconazole (Sigma) was used as a positive control drug for antifungal susceptibility testing. After the incubation at 35°C for 24 h, the plates were read with the microplate reader to determine MIC values for the *Candida* strains. For MFC, 10 μL of the suspension from the first six wells were inoculated on the PDA plates as a control for yeast viability. Following incubation (35°C/24 h), the lowest concentration of the test compound at which no visible growth on the plate was observed was determined as MFC [15]. All experiments were carried out in triplicate. Data regarding the MIC values of all five tested compounds against the bacterial and fungal strains were analyzed using one-way analysis of variance (ANOVA) with Tukey's multiple comparison test. All analyses were carried out using the SigmaPlot Version 15 (15.0.0.13) software (Systat Software Inc., San Jose, CA). A *p*-value of less than 0.05 was considered statistically significant.

### 3.3. DNA Cleavage Assay

The DNA cleavage activity of the test compounds was studied by using agarose gel electrophoresis, and the ability of the compound to cleave was evaluated using plasmid pBR322 DNA (Thermo Scientific, SD0041, 0.5 μg/mL) [33]. In the experiments, 0.3 μg of supercoiled plasmid pBR322 DNA was treated with four different concentrations of 125, 250, 500, and 1000 μM of each compound and then incubated in the dark for 24 h at 37°C. After the incubation period, the samples were mixed with loading buffer and then loaded on 1% agarose gels containing 0.5 μg/mL ethidium bromide and electrophoresed in TBE buffer for 1 h at 70 V. Gels were visualized with a UV transilluminator (DNR Minilumi Bio-imaging Systems Ltd., Jerusalem, Israel). At least three independent DNA cleavage experiments were performed.

### 3.4. Process Steps in Biosensor Design

Carbon paste electrodes modified with 5 different Schiff bases containing 4-aminoantipyrine were prepared and used in glucose biosensor design. For this purpose, the following procedures were carried out, respectively.

a. Firstly, modified carbon paste electrodes (MCPEs) were prepared with synthesized materials (4AA-Fc, 4AA-SA, 4AA-FSA, 4AA-ClFSA, 4AA-FMeSA).
b. In the literature, the principle of glucose biosensors is generally based on the measurement of oxidation currents of H_2_O_2_ which is released as a result of the reaction between glucose and GOx enzyme [34]. Based on this principle, the sensitivity of 5 MCPEs to hydrogen peroxide was determined.
c. The most sensitive of the modified electrodes to H_2_O_2_ was selected and used in glucose biosensor design.
d. The best potential and the best Schiff base amount were determined by measuring the anodic currents of H_2_O_2_ with the selected MCPE.
e. The biosensor was prepared by immobilizing GOx enzyme to the optimized MCPE.
f. The effect of the amount of glutaraldehyde (GL) used in immobilization processes on the sensitivity of the biosensor to glucose was investigated.
g. The best working pH and temperature of the biosensor were determined.
h. The effect of substrate concentration on the response of the prepared biosensor to glucose and the calibration graph were determined.
i. Finally, reproducibility, storage stability, and interference studies, which are the factors affecting the performance of the biosensor, were carried out.

### 3.5. Preparation of MCPE and Enzyme Immobilization

**Figure 3:**
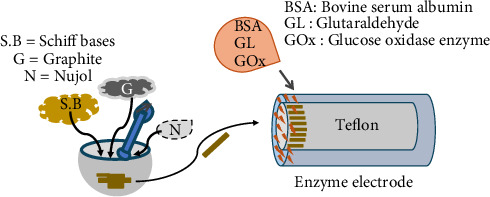
Preparation of glucose biosensor.

The preparation of the glucose biosensor is schematized in Figure 3. The carbon paste electrode was cleaned with distilled water. 62.5 mg graphite powder and 95 μL mineral oil (nujol) were mixed to form a paste. Then, the carbon paste was filled into the electrode. The MCPE contained Schiff base in addition to graphite powder and nujol.

A mixture of 2.0 mg bovine serum albumin (BSA), 50 μL phosphate buffer (pH 7.0), 30 μL GL, and 50 μL GOx (1000 units/mL) enzyme was dropped onto the prepared MCPE surface, and the electrode was dried at room temperature. The electrode was then washed with distilled water to remove the enzyme that could not be immobilized to the surface. When not in use, they were stored in phosphate buffer at +4°C in the refrigerator.

### 3.6. Amperometric Measurements

**Figure 4:**
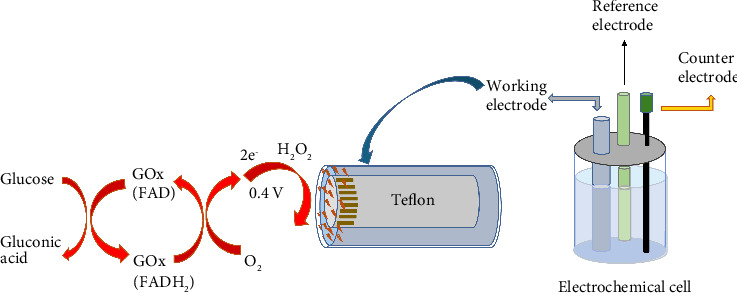
Reaction for glucose determination.

The electrochemical measurement is schematized in Figure 4. The enzyme GOx catalyzes the oxidation of glucose in oxygenated medium to form gluconic acid and H_2_O_2_. Glucose determination was based on the measurement of oxidation currents at 0.4 V of H_2_O_2_ formed at the end of the enzymatic reaction [35, 36]. The prepared MCPE was equilibrated at 0.4 V in a cell containing 1 mL sodium chloride (1M) and 9 mL phosphate buffer (pH 7.0). Then glucose solution was added, and the solution was stirred for a while and measured after 200 s. This measured current value was subtracted from the equilibrium current, and the current difference (Δ*i*) value was calculated.

### 3.7. In Silico Studies (ADME Profiling and Cytotoxicity Prediction)

SwissADME (https://www.swissadme.ch/index.php) was used to determine the pharmacokinetics and drug similarity properties of Schiff bases in silico [37]. Prediction of the cytotoxic effects of molecules on cancer cell lines was performed with Cell Line Cytotoxicity Predictor (https://www.way2drug.com/cell-line/) [25].

## 4. Results and Discussion

Schiff bases obtained by the condensation of 4-aminoantipyrine and substituted salicylaldehydes (5-fluorosalicylaldehyde, 3-chloro-5-fluorosalicylaldehyde, 5-fluoro-3-methylsalicylaldehyde) and ferrocenecarboxaldehyde were first synthesized for this research. Therefore, after the characterization of Schiff bases derived from 4-aminoantipyrine, findings on antimicrobial activity, DNA cleavage, and glucose biosensor design are given in detail.

### 4.1. Characterization

**Figure 5:**
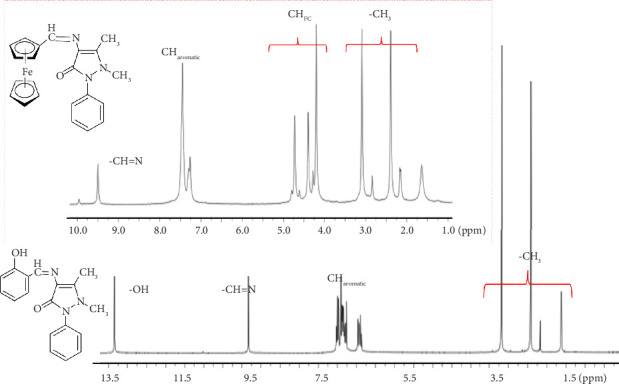
^1^H-NMR spectra of (4AA-Fc) and (4AA-SA).

The Schiff bases were soluble in polar organic solvents but not in apolar solvents (like CCl_4_, benzene, and hexane). As seen in Table 1, the elemental analysis results are in agreement with the proposed structures. Table 1 summarizes the main ^1^H-NMR, IR bands, chemical, and physical properties of the Schiff bases. ^1^H-NMR spectra are given in Figure 5 for 4AA-Fc and 4AA-SA.

When the IR spectra are evaluated, 3100–3050 cm^−1^ and 3058–3000 cm^−1^ may be the vibrational frequencies of -OH and aromatic -CH groups, respectively [38]. The peaks at 2970–2870 cm^−1^ and 1652–1648 cm^−1^ are for aliphatic -CH_3_ and C=O stretching, respectively [39]. The *ν* (CH=N) stretching frequencies were observed in the range of 1600 and 1590 cm^−1^ [40]. In addition, the characteristic peaks at 1457, 1410 and 1378 cm^−1^ of ferrocene were observed for compound (4AA-Fc) [41]. This is an expected result.

In UV spectra of Schiff bases, multiple bands emerged between 250 and 300 nm in the UV region (Table 2). Between 250 and 300 nm bands were attributed to the σ ⟶ *σ*^∗^ and *n* ⟶ *σ*^∗^ transition of the aromatic ring. The bands at 301–364 nm correspond to the -C=O and -C=N transitions of groups *π* ⟶ *π*^∗^ [42]. In the UV–GB spectra of the organometallic group, (*d* ⟶ *d*) transitions of ferrocene were observed at 468 nm.

The protons observed in the ^1^H-NMR spectra of Schiff bases appeared as expected (Figure 5, Table 1). The appearance of the characteristic multiple peaks belonging to the aromatic ring in the range of 7.20–7.75 ppm was considered as evidence that the aldehyde was incorporated into the 4-AAP [43, 44]. It was predicted that the chemical shift of Schiff bases containing an -OH group may be between 13.50 and 15.50 ppm [42]. It was suggested that the chemical shifts at 9.50 and 9.80 ppm correspond to -CH=N protons. In the ferrocene-attached Schiff base, specific protons of ferrocene were observed as indicated in the references. The single peak at 4.24 ppm is due to the Cp ring to which 5 hydrogen atoms are attached. In addition, the peaks at 4.63 and 4.42 ppm are due to protons with hydrogens in two chemical environments to which the imine group is attached and three bonds away [45, 46]. Among the methyl groups in 4-aminoantipyrine, the group close to -CH=N appeared in the higher area, as expected [44]. (4AA-SA), (4AA-FSA), (4AA-ClFSA), and (4AA-FMeSA) have aromatic protons at 6.90–6.75 ppm, 7.05–7.20 ppm, 7.00–6.90 ppm, and 6.90–6.80 ppm, respectively.

### 4.2. Antimicrobial Activity

**Figure 6:**
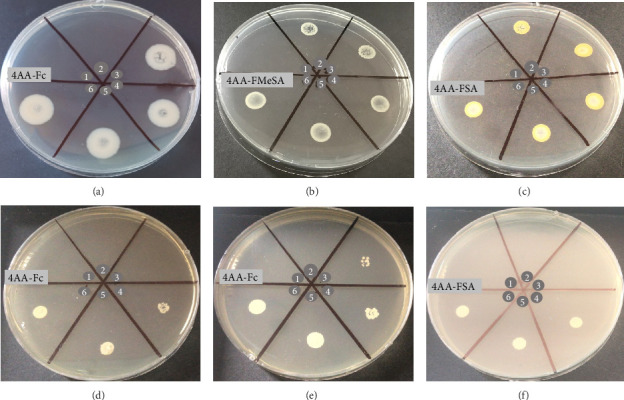
Minimum bactericidal concentration of compounds against *B. cereus* ATCC 14579, *Salmonella* Typhimurium ATCC 14028, and *S. aureus* ATCC 6538 in a, b, and c, respectively. Minimum fungicidal concentration of compounds against *C. albicans* ATCC 10231, *C. krusei* ATCC 6258, and *C. parapsilosis* ATCC 22019 in d, e, and f, respectively. The numbers 1, 2, 3, 4, 5, and 6 show compound concentrations of 1000, 500, 250, 125, 62.5, and 31.25 μM, respectively.

The minimum inhibitor concentration (MIC) results of compounds against bacteria and fungi are exhibited in Tables 3 and 4, respectively. The MBC and MFC of compounds are presented in Figure 6. All compounds showed antibacterial activity against reference strains at similar rates. MIC values were in the range of 125–500 μM against Gram-positive bacteria, but 250–500 μM for Gram-negative bacteria.

Generally, 4AA-Fc and 4AA-ClFSA compounds exhibited more antibacterial activity than others against all Gram-positive and Gram-negative bacteria according to low MIC values. Interestingly, the compound 4AA-SA and the compound 4AA-FSA were more effective against Gram-negative bacteria than Gram-positive bacteria compared to MIC values. *Streptococcus pyogenes* ATCC 19615 was the most sensitive at concentrations of 125 μM against the tested compounds among all bacterial strains. Statistical analysis of MIC data of all tested compounds against all bacterial strains, including Gram-positive and Gram-negative bacteria, revealed that there was no statistically significant difference between the MIC values of all compounds against Gram-positive and Gram-negative bacteria (*p*=0.068). Furthermore, there was no statistically significant difference in the MIC values of all compounds between Gram-positive bacteria (*p*=0.122). However, the difference in MIC values of all compounds against the Gram-negative bacteria was statistically significant (*p*=0.039). The MBC is the lowest agent concentration where 99.9% of the bacteria were killed on the subculture. The ratio MBC/MIC ≤ 4 was considered as the bactericidal effect, but the ratio MBC/MIC > 4 was defined as the bacteriostatic effect [47, 48]. We evaluated antibacterial effect based on the MBC/MIC ratio (Table 3). The compounds 4AA-Fc, 4AA-FSA, 4AA-ClFSA, and 4AA-FMeSA displayed bactericidal activity against all tested Gram-positive bacteria except for *B. subtilis, B. thuringiensis*, and *S. aureus* ATCC 25923. However, the compound 4AA-SA exhibited bactericidal activity against only four Gram-positive bacterial strains (*L. monocytogenes* RSKK 475, *B. cereus* ATCC 14579, *S. epidermidis* ATCC 12228, and *S. pyogenes* ATCC 19615) (Table 3). However, all compounds had bactericidal effect against all tested Gram-negative bacteria except *E. coli* ATCC 8739. The compound 4AA-Fc was the most potent compound with low MBC/MIC ratios for bactericidal effect which kills both Gram-positive and Gram-negative pathogens (Table 3).

The MIC, the MFC, and MFC/MIC values for all compounds tested against fungal strains are shown in Table 4. The MIC of compounds including 4AA-Fc, 4AA-SA, 4AA-FSA, and 4AA-FMeSA was in the range of 125–250 μM. A statistically significant difference (*p*=0.036) was observed in the MIC values of all compounds between the *Candida* strains. The MIC of the compound 4AA-ClFSA was in the range of 62.5–250 μM but had high MFC values (> 1000). The compound 4AA-Fc had a MIC of 125 μM for all *Candida* strains except *C. krusei*, indicating that it was the most effective compound with low MIC and MFC values. Notably, the compound 4AA-Fc exhibited significantly greater antifungal activity against most *Candida* strains than fluconazole, compared to the MIC values. According to MFC/MIC ratios, the compound 4AA-Fc, 4AA-SA, 4AA-FSA, and 4AA-FMeSA had fungicidal activity against these pathogenic *Candida* strains. However, the compound 4AA-ClFSA showed fungistatic activity against the test strains due to high MFC/MIC ratios (Table 4). In addition, a few example plates obtained for MBC and MFC of the compounds are illustrated in Figure 6.

In previous studies, the antimicrobial activities of Schiff base derivatives of 4-aminoantipyrine were documented [10, 11, 18]. Aguilar-Llanos et al. synthesized eight Schiff bases by the reaction of 4-aminoantipyrine and tested their antibacterial and antifungal activity [11]. They found that all Schiff base derivatives against *P. aeruginosa* ATCC 27853 had MIC values above 250 μM. According to our findings, the MIC values for all Schiff bases from 4-aminoantipyrine against *P. aeruginosa* ATCC 14028 were mostly 250 μM. Compared with our antifungal results, Aguilar-Llanos et al. observed similar MIC results for *C. albicans* [11]. Moreover, the MIC values of the compounds, including Schiff base from 4-aminoantipyrine, demonstrated a higher antibacterial effect against Gram-positive strains than against Gram-negative strains [18]. Several synthesized Schiff base derivatives of 4-aminoantipyrine were tested against selected bacteria, fungi, and Leishmania parasites. Results showed that different compounds had specific activities against specific microorganisms [10].

The variation in the activity of different studied compounds, such as Schiff base derivatives of 4-aminoantipyrine, against different microorganisms may depend on the impermeability of the cells of microorganisms to the compounds, the difference in ribosomes of microbial cells, and the difference in cell wall structure and composition among the microbes [10, 18, 49].

### 4.3. DNA Cleavage

**Figure 7:**
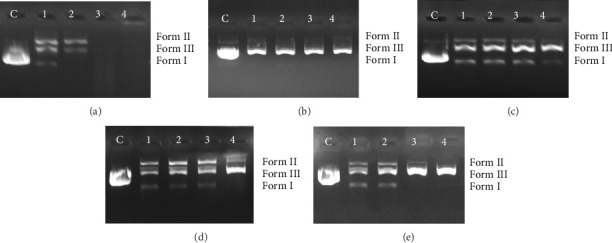
Agarose gel electrophoresis images of pBR322 plasmid DNA incubated at 37°C for 24 h with increasing concentrations of (a) 4AA-Fc, (b) 4AA-SA, (c) 4AA-FSA, (d) 4AA-ClFSA, and (e) 4AA-FMeSA. C: Control—supercoiled plasmid DNA; lanes 1–4: 125, 250, 500, and 1000 μM concentrations of compounds, respectively.

The DNA cleavage studies on pBR322 revealed that all compounds induced efficient DNA cleavage in the dark without the addition of an external oxidant or reductant (Figure 7). The DNA cleavage propensity was examined by following the degree of conversion of the supercoiled DNA form (Form I) to nicked circular form (Form II) and linear form (From III). It is important to note that the three types of DNA, as described, migrate along the agarose at varying rates in the following order: Form I > Form III > Form II [50]. All the Schiff bases including (4AA-Fc) coded Schiff base also showed a good self-activating DNA cleavage activity at low concentration (125 μM). However, the compound 4AA-Fc had the most efficient activity, showing a complete DNA degradation. As shown in Figure 7(a), at a 125 μM concentration of 4AA-Fc, supercoiled plasmid DNA (Form I) was converted into nicked circular DNA (Form II, one of the two strands was cut) and linear DNA (Form III, both strands were cut). At a concentration of 250 μM, Form I completely disappeared, and the nicked DNA (Form II) and the linear DNA (Form III) were observed. Interestingly, at concentrations of 500 and 1000 μM, the compound 4AA-Fc showed 100% DNA destruction, indicating strong cleavage activity (Figure 7(a), lanes 3 and 4). The 4AA-SA compound converted the supercoiled plasmid DNA (Form I) into linear DNA (Form III) at all concentrations tested (125–1000 μM), suggesting a concentration-independent cleavage effect, as shown in Figure 7(b). At the first three concentrations (125, 250, and 500 μM) of the 4AA-FSA and 4AA-ClFSA compounds, supercoiled plasmid DNA (Form I) was converted into nicked circular DNA (Form II) and linear DNA (Form III), and then at the 1000 μM concentration of these compounds, linear DNA was observed (Figures 7(c) and 7(d)). At 125 and 250 μM concentrations of the 4AA-FMeSA, there was an increase in nicked circular DNA (Form II) and a decrease in supercoiled plasmid DNA (Form I). At 500 and 1000 μM concentrations of this compound, while the supercoiled plasmid DNA (Form I) had totally vanished, linear DNA (Form III) was generated (lanes 3-4 in Figure 7(e)).

Previous studies based on the results DNA cleavage activity emphasized that the different DNA cleavage efficiency of the synthesized compounds may be due to the different binding affinities of the compounds to DNA [13, 23, 51]. Besides, researchers report that DNA cleavage activity is a useful tool for designing biosensors and developing therapeutic agents. It is also a crucial property for the potential of DNA-targeted cancer drugs [19, 20].

### 4.4. Design of Glucose Biosensor

#### 4.4.1. Determination of the Response of CP and MCP Electrodes to H_2_O_2_, Working Potential, and the Amount of 4AA-Fc

**Figure 8:**
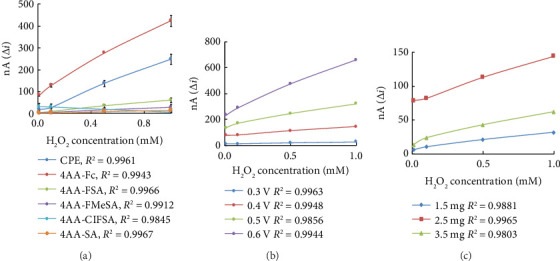
(a) Amperometric responses of CPE and MCPE to different concentrations of H_2_O_2_, (b) Effect of working potential on H_2_O_2_ response of MCPE, (c) Effect of 4AA-Fc amount on H_2_O_2_ response of MCPE.

Determination of the response of CP and MCP electrodes to H_2_O_2_ was performed by measuring the anodic response currents of H_2_O_2_. Five newly synthesized and characterized Schiff bases (4AA-Fc, 4AA-SA, 4AA-FSA, 4AA-ClFSA, and 4AA-FMeSA) were used to modify the CP electrode. CP and MCP electrodes were brought to constant current at +0.7 V potential, and equilibrium current was recorded. H_2_O_2_ concentration was added to the cell in the range of 1.0 × 10^−5^–1.0 × 10^−3^ M, and the response currents obtained against H_2_O_2_ concentration were graphed (Figure 8(a)). When the graph was analyzed, it was seen that the best response current belonged to the MCP electrode containing 4AA-Fc. The response currents of the other Schiff bases to H_2_O_2_ were lower than the response currents of the CP electrode. Therefore, the carbon paste electrode modified with 4AA-Fc was used in subsequent studies. The design of the glucose biosensor was done with this material. 4AA-Fc has a ferrocene structure. Since ferrocene facilitates electron transfer, it is thought to generate higher response currents than other Schiff bases.

Anodic currents of H_2_O_2_ at different potentials (+0.7, +0.6, +0.5, +0.4, +0.3 V) were measured with a MCPE. The measured current difference (Δi) values against H_2_O_2_ concentration were plotted.  Figure 8(b) shows that as the potential increases, the anodic currents of hydrogen peroxide also increase. It is stated in the literature that working at high potentials creates interference problems when working with real samples [36]. Therefore, +0.4 V with good linearity was chosen as the working potential. Subsequent studies were carried out at +0.4 V. The line at +0.3 V also had a good correlation coefficient, but since it showed low currents, this potential was not chosen as the working potential.

In order to investigate the effect of 4AA-Fc amount on the amperometric response of H_2_O_2_, MCPE was prepared using 1.5, 2.5, 3.5 mg 4AA-Fc. MCPE was brought to constant current at +0.4 V, and the equilibrium current was recorded. H_2_O_2_ concentration was added to the cell in the range of 1.0 × 10^−5^–1.0 × 10^−3^ M, and the response currents obtained against H_2_O_2_ concentration were graphed (Figure 8(c)). Figure 8(c) shows that when the amount of 4AA-Fc was increased from 1.5 to 2.5 mg, the response currents increased and when it was increased to 3.5 mg, the response currents decreased. When 3.5 mg 4AA-Fc was used, it was observed that the mechanical stability of the prepared carbon paste electrode was not good, and the paste pieces were separated from the surface. Therefore, 2.5 mg of 4AA-Fc was used in future studies.

#### 4.4.2. Effect of GL Amount, pH, and Temperature on the Response of Biosensor

**Figure 9:**
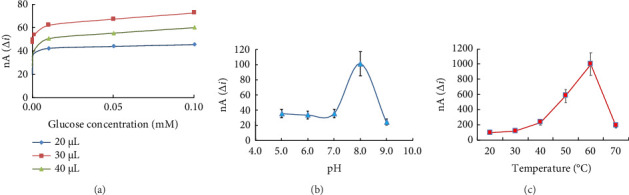
(a) Effect of glutaraldehyde amount on glucose response of MCPE (2.5% glutaraldehyde), (b) Effect of pH on glucose response of MCPE, (c) Effect of temperature on glucose response of MCPE.

GL is one of the most preferred cross-linking reagents in studies. However, the use of GL can reduce the activity of the enzyme, so the amount of GL must be optimized [52]. To determine the amount of GL used in the prepared enzyme electrode, biosensors were prepared with 20.0, 30.0, 40.0 μL GL (2.5%). Currents read against 1.0 × 10^−8^–1.0 × 10^−3^ M glucose concentrations were plotted (Figure 9(a)). It was observed that the response currents increased when the amount of GL was increased from 20.0 to 30.0 μL; however, the currents decreased when the amount was increased to 40.0 μL.

The results were interpreted as follows; it was thought that 20.0 μL of GL was not enough to immobilize the enzyme and could not hold the structure together, while 40.0 μL of GL was too much and the enzyme lost activity as a result of excessive binding [53]. The highest current value was found at the electrode using 30 μL GL, and subsequent studies were performed with 30 μL GL.

In order to investigate the effect of pH on the amperometric response current of the prepared glucose biosensor, buffer solutions with five different pH values were prepared. Acetic acid–sodium acetate buffer solution was used for pH 5.0; phosphate buffer solution (Na_2_HPO_4_-NaH_2_PO_4_) was used for 6.0, 7.0, 8.0; and glycine buffer solution was used for 9.0. With the glucose biosensor, the anodic currents of H_2_O_2_ released as a result of the enzymatic reaction at a concentration of 1.0 × 10^−4^ M glucose at +0.4 V were measured, and the currents were plotted against pH.  Figure 9(b) shows that the maximum activity was observed at pH 8.0. pH values of 7.4 [54], 8.0 [55, 56] are encountered in the literature. The different carriers used during the immobilization of the enzyme cause the pH to change.

Since enzymes have a protein structure, they are affected by temperature changes. At low temperatures, the activity of enzymes is low, while at high temperatures the protein structure of the enzyme denatures and the activity decreases. Enzymes have their own optimum temperatures at which they show maximum activity. The effect of temperature on the amperometric response of biosensor prepared at 20°C, 30°C, 40°C, 50°C, 60°C, 70°C on glucose was investigated. The anodic currents of H_2_O_2_ released as a result of the enzymatic reaction with the glucose biosensor at a concentration of 1.0 × 10^−4^ M glucose at +0.4 V were measured, and the currents were plotted against temperature.  Figure 9(c) shows that the maximum current was observed at 60°C. However, in the following studies, room temperature was used for more practical and easy measurements. Optimum temperatures for glucose biosensors prepared with various substances in literature research may differ. The operating temperature of GOx was reported to be around 55°C and 30°C [56, 57]. Another study observed a decrease in current values at temperatures higher than 40°C and took the temperature as 25°C [58]. In another literature, the maximum temperature value was found to be 60°C [59].

#### 4.4.3. Effect of Substrate Concentration and Calibration Curves, and Operational and Storage Stability of the Biosensor

**Figure 10:**
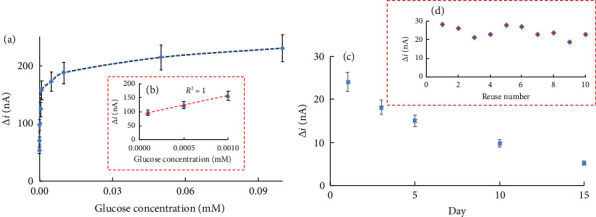
(a) Effect of glucose concentration on the response of biosensor (Michael–Menten curve), (b) effect of glucose concentration on the response of biosensor (calibration curve), (c) storage stability of the biosensor (+0.4 V potential, 0.1 M pH 8.0 phosphate buffer at 25°C), (d) operational stability of the biosensor (+0.4 V potential, 0.1 M pH 8.0 phosphate buffer at 25°C).

To examine the effect of glucose concentration on the response of the biosensor, the anodic currents of H_2_O_2_ formed as a result of the enzymatic reaction of 1.0 × 10^−8^–1.0 × 10^−3^ M at increasing glucose concentrations at +0.4 V with the enzyme electrode were recorded and the current differences versus glucose concentration were plotted (Figure 10(a)). When the Figure was examined, it was seen that there was a linear operating range in the range of 1.0 × 10^−7^–1.0 × 10^−6^ M (*R*^2^ = 1.0), and the lower limit of determination was 1.0 × 10^−8^ M (Figure 10(b)). Km (observed) and Imax constants for GOx enzyme immobilized on 4AA-Fc were calculated as 0.0149 μM and 149.25 nA, respectively. The Km value obtained shows the enzyme's affinity for the substrate. The Km values given in the literature for GOx enzyme are 2.97, 11.9, and 18.0 mM [59–61]. Imax values given for GOD is 0.097 μA [59]. Compared to the literature, we can say that the linear operating range of our glucose biosensor is at low concentrations and the limit of detection is also low. As it is known, in electrochemical biosensors, the determination of substances at low concentrations and low determination limit increases the value of the biosensor.

In order to examine the operational stability of the prepared glucose biosensor, the anodic current of H_2_O_2_ released as a result of the enzymatic reaction at a glucose concentration of 1.0 × 10^−6^ M at +0.4 V with MCPE was measured. This study was repeated 10 times by renewing the cell. Current differences were plotted against the number of measurements (Figure 10(d)). At the end of 10 measurements, it was determined that the biosensor lost 21.73% of its initial activity. The relative standard deviation was calculated as 12.70% from the current changes obtained. To investigate the storage stability of the prepared glucose biosensor, currents were measured on different days (1, 3, 5, 10, and 15) at a glucose concentration of 1.0 × 10^−6^ M at +0.4 V and plotted against the number of days (Figure 10(c)). It was observed that the biosensor retained 20.84% of its initial activity after 15 days.

#### 4.4.4. Interference Effects for the Biosensor

To study the effect of interfering species on glucose determination, 3.0 × 10^−4^ M uric acid and 1.0 × 10^−4^ M ascorbic acid, 1.0 × 10^−4^ M paracetamol, and 2.5 × 10^−3^ M urea were used [46]. Glucose concentration was kept constant at 1.0 × 10^−6^ M, and these substances were added into the working cell. The change in current value was determined. As a result, it was determined that uric acid and urea had no interference effect, ascorbic acid interfered 2.33%, and paracetamol 4.35%.

### 4.5. In Silico Analysis (ADME Profiling and Cytotoxicity Prediction)

The ADME parameters of the synthesized compounds were evaluated pharmacokinetically. We used the ADME profile to determine whether the compound we synthesized had pharmacokinetic properties suitable for the drug and whether it had properties that would cause safety concerns in humans [62]. The estimated results of cell line cytotoxicity of biocompatible aminoantipyrine-derived Schiff bases evaluated for their ADME properties by the in silico approach are given in Tables 5 and 6.

The pharmacological effects and biological activities of four compounds were analyzed through the Cell Line Cytotoxicity Predictor online server. The values of Pa and Pi vary between 0.004 and 0.894. If Pa > 0.7, the probability of experimental pharmacological action is high. If 0.5 < Pa < 0.7, the probability of experimental pharmacological action is less [63]. All of the aminoantipyrine-derived Schiff bases exhibit excellent Pa and Pi values and show cytotoxicity against the HOP-62 non-small cell lung carcinoma cell line (except 4AA-Fc). This suggests that Schiff bases with significant bioavailability scores possess favorable drug-like properties, indicating their potential for development as therapeutic drugs.

The ADME study using SwissADME revealed that all compounds had high gastrointestinal (GI) absorption. All Schiff bases were able to pass through the blood–brain barrier (BBB), which protects the central nervous system by separating the brain tissues from the bloodstream [37]. The results indicated that all compounds were predicted to be nonsubstrates of P-gp (P-glycoprotein). This protein acts as a drug-extracting pump that requires energy to function and plays a crucial role in multidrug resistance by facilitating the active transfer of anticancer drugs from the intracellular to the extracellular compartment [37]. Cytochrome P450 (CYP) enzymes are involved in the metabolism of the majority of drugs and occur mainly in the liver and intestine. They are essential to determine the effect of the response of an anticancer drug [37]. In this research, Schiff bases (4AA-FMeSA) and (4AA-ClFSA) inhibited liver enzymes (CYP1A2, CYP2C19, CYP2C9, and CYP2D6), whereas Schiff bases coded (4AA-Fc), (4AA-SA), and (4AA-FSA) did not. This result makes it possible to better evaluate (4AA-Fc), (4AA-SA), and (4AA-FSA) coded Schiff bases as drugs.

## 5. Conclusion

Novel 4-aminoantipyrine-derived Schiff bases ((4AA-Fc), (4AA-SA), (4AA-FSA), (4AA-ClFSA), and (4AA-FMeSA)) were synthesized, characterized, and investigated for their antimicrobial and anticancer activities, in silico prediction of ADME and cytotoxicity properties, and glucose biosensing application. All novel Schiff bases had good antibacterial and antifungal activities. The difference in the MIC values of all compounds against the Gram-negative bacterial and fungal strains was statistically significant. Moreover, all compounds exhibited self-activating DNA cleavage ability at low concentrations without any external agents. In addition, 4AA-Fc showed the most efficient activity with a complete DNA degradation. DNA cleavage has a crucial role in variety of biological applications in genome editing, disease treatment, and biosensor design. 4AA-Fc coded Schiff base which showed the best sensitivity to H_2_O_2_ was used in glucose biosensor design. The prepared biosensor was observed to have a low concentration operating range (1.0 × 10^−7^–1.0 × 10^−6^ M (*R*^2^ = 1.0)) and very low detection limits (1.0 × 10^−8^ M). As it is known, in electrochemical biosensors, the determination of substances at low concentrations and low determination limits increases the value of the biosensor. The sensitivity and accuracy of a biosensor for glucose detection can also be impaired by microorganisms in the external environment. Therefore, there is a great need for the development of biosensors with antibacterial and antifungal properties. *In silico* cell line cytotoxicity studies revealed that Schiff bases with significant bioavailability scores possess favorable drug-like properties, indicating their potential for development as therapeutic drugs. Furthermore, Schiff bases were predicted to have good ADME properties, especially in terms of Lipinski's rule and GI absorption. In addition, the compounds were not a P-gp substrate (p-glycoprotein). Thus, they could be a potential anticancer agent. The pharmacokinetic and cell line cytotoxicity prediction results presented here may be useful for further studies on new biologically active substances. In conclusion, this study may contribute to the synthesis of antimicrobial or anticancer agents, or the design of biosensors based on 4-aminoantipyrine-derived Schiff bases.

## Figures and Tables

**Table 1:** Analytical data, some of the physical properties, IR (cm^−1^) and ^1^H-NMR (ppm) spectra data of Schiff bases.

Symbol Formula (*M*_*w*_, g·mol^−1^), yield (%) color/m.p (°C)	Elemental analysis found (calculated) %			FTIR (cm^−1^) ν_OH_, ν_ArH_ ν_-CH3_, ν_C=O_ ν_C=N_, ν_Fc_	^1^H-NMR (DMSO-d_6_, ppm) -CH=N, -OH R = Fc, SA, FSA, ClFSA FMeSA	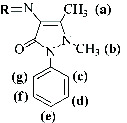
	C	H	N			
(4AA-Fc) C_22_H_21_N_3_OFe (399.10), 72.8 orange/208	65.31 (66.18)	5.30 (5.30)	10.99 (10.52)	—/3058 2970/1648 1590, 1457	9.50, — 7.51–7.20_(c–g)_, 4.75–4.10_(R)_, 3.12_(b)_, 2.13_(a)_	

(4AA-SA) C_18_H_17_N_3_O_2_ (307.13), 71.2 yellow/196.5	68.97 (70.34)	5.58 (5.58)	18.09 (13.67)	3100/3056 2970/1652 1591	9.55, 13.30 7.75–7.20_(c–g)_, 6.90–6.75_(R)_, 3.10_(b)_, 2.30_(a)_	

(4AA-FSA) C_18_H_16_N_3_FO_2_ (325.12), 66.4 light yellow/202	66.22 (66.45)	5.09 (4.96)	14.18 (5.84)	3100/3052 2970/1650 1600	9.56, 13.10 7.50–7.20_(c–g)_, 7.05–7.20_(R)_, 3.10_(b)_, 2.40_(a)_	

(4AA-ClFSA) C_18_H_15_N_3_ClFO_2_ (359.08), 67.0 yellow/170	60.49 (60.09)	4.49 (4.20)	13.12 (11.68)	3100/3058 2870/1650 1601	9.75, 13.40 7.50–7.05_(c–g)_, 7.00–6.90_(R)_, 3.25_(b)_, 2.45_(a)_	

(4AA-FMeSA) C_19_H_18_N_3_FO_2_ (339.14), 65.7 light yellow/175	67.12 (67.24)	5.49 (5.35)	13.81 (12.38)	3150/3000 2900/1650 1596	9.80, 13.40 7.60–7.20_(c–g)_, 6.90–6.80_(R)_, 3.30_(b)_, 2.50_(a)_	

**Table 2:** UV-GB spectra data of Schiff bases.

**Symbol**	**Transitions** ***λ***_max_ ** , nm, Abs.**			
	**(*σ* ⟶ ** **σ** ^∗^ ** )** **(*n* ⟶ ** **σ** ^∗^ ** )**	(***π*** − ***π***^∗^)_Fc_ (***π*** − ***π***^∗^)_arom_	(**d** − **d**)_Fc_	

(4AA-Fc)	288 (2.48) 294 (2.24)	328 (2.51) 314 (2.48)	466 (0.21)	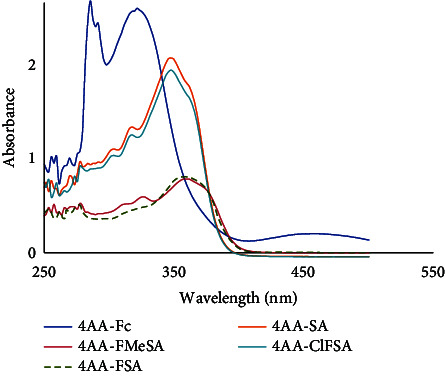
(4AA-SA)	286 (0.89) 308 (1.03)	— 324–364 (1.22–1.59)	—	
(4AA-FSA)	280 (0.409) 328 (0.466)	— 364–376 (0.78–0.71)	—	
(4AA-ClFSA)	284 (0.71) 300 (0.99)	— 322–358 (1.03–1.42)	—	
(4AA-FMeSA)	280 (0.372) 314 (0.407)	— 332–376 (0.48–0.55)	—	

**Table 3:** Minimal inhibitory concentration (MIC) and minimal bactericidal concentration (MBC) of chemicals against Gram‐positive and Gram-negative reference bacteria strains used in this study.

Reference Bacterial strain	4AA-Fc			4AA-SA			4AA-FSA			4AA-ClFSA			4AA-FMeSA			Ampicillin	
	MIC × 10^2^	MBC × 10^2^	*R*	MIC × 10^2^	MBC × 10^2^	*R*	MIC × 10^2^	MBC × 10^2^	*R*	MIC × 10^2^	MBC × 10^2^	*R*	MIC × 10^2^	MBC × 10^2^	*R*	MIC × 10^2^	MBC × 10^2^
Gram-positive																	
*L. monocytogenes* RSKK 475	2.5	10.0	4	5.0	10.0	2	5.0	10.0	2	2.5	10.0	4	2.5	10.0	4	0.078	0.078
*B. cereus* ATCC 14579	2.5	5.0	2	2.5	10.0	4	5.0	10.0	2	2.5	10.0	4	2.5	10.0	4	0.625	0.125
*B. subtilis*	2.5	> 10.0	> 4	5.0	> 10.0	nd	2.5	> 10.0	> 4	2.5	> 10.0	> 4	5.0	> 10.0	nd	0.625	2.5
*B. thuringiensis*	2.5	> 10.0	> 4	5.0	> 10.0	nd	2.5	> 10.0	> 4	2.5	> 10.0	> 4	2.5	> 10.0	> 4	10.0	10.0
*S. aureus* ATCC 6538	2.5	10.0	4	5.0	> 10.0	nd	5.0	10.0	2	2.5	10.0	4	5.0	10.0	2	0.039	0.039
*S. aureus* ATCC 25923	5.0	> 10.0	nd	5.0	> 10.0	nd	5.0	> 10.0	nd	2.5	> 10.0	> 4	5.0	> 10.0	nd	0.039	0.078
*S. aureus* ATCC 29213	5.0	10.0	2	5.0	> 10.0	nd	5.0	10.0	2	5.0	10.0	2	5.0	10.0	2	0.0195	0.0195
*S. epidermidis* ATCC 12228	5.0	10.0	2	2.5	10.0	4	5.0	10.0	2	2.5	10.0	4	2.5	10.0	4	0.039	0.039
*S. pyogenes* ATCC 19615	1.25	1.25	1	2.5	5.0	2	1.25	1.25	1	1.25	2.5	2	1.25	5.0	4	0.039	0.039
Gram-negative																	
*E. coli* ATCC 8739	5.0	> 10.0	nd	2.5	10.0	4	5.0	10.0	2	2.5	10.0	4	5.0	10.0	4	0.1562	0.625
*E. coli* ATCC 25922	5.0	10.0	2	5.0	10.0	2	2.5	10.0	4	2.5	10.0	4	5.0	10.0	4	0.1562	0.1562
*E. cloacae* NCIMB 13597	5.0	10.0	2	5.0	10.0	2	2.5	10.0	4	2.5	10.0	4	5.0	10.0	4	10.0	10.0
*E. cloacae* ATCC 23355	2.5	10.0	4	2.5	10.0	4	2.5	10.0	4	2.5	10.0	4	2.5	10.0	4	0.625	2.5
*K. pneumoniae* ATCC 700603	2.5	10.0	4	5.0	10.0	2	2.5	10.0	4	2.5	10.0	4	2.5	10.0	4	0.1562	0.3125
*P. aeruginosa* ATCC 27853	2.5	10.0	4	2.5	10.0	4	2.5	10.0	4	5.0	10.0	2	5.0	10.0	2	10.00	10.0
*Salmonella* Typhimurium ATCC 13311	5.0	10.0	2	2.5	10.0	4	5.0	10.0	2	2.5	10.0	4	5.0	10.0	4	0.039	0.039
*Salmonella* Typhimurium ATCC 14028	5.0	10.0	2	2.5	10.0	4	2.5	10.0	4	2.5	10.0	4	5.0	10.0	4	0.1562	0.1562
*Salmonella* Enteritidis ATCC 13076	5.0	10.0	2	2.5	10.0	4	2.5	10.0	4	2.5	10.0	4	2.5	10.0	4	0.039	0.039

*Note:* MIC and MBC data were expressed as µM. *R*; MBC/MIC ratio ≤ 4 means bactericidal activity and > 4 means bacteriostatic activity.

Abbreviation: nd, not determined.

**Table 4:** Minimal inhibitory concentration (MIC) and minimal fungicidal concentration of chemicals against reference *Candida* strains.

Strain	4AA-Fc			4AA-SA			4AA-FSA			4AA-ClFSA			4AA-FMeSA			Fluconazole	
	MIC 10^2^	MFC 10^2^	*R*	MIC 10^2^	MFC 10^2^	*R*	MIC 10^2^	MFC 10^2^	*R*	MIC 10^2^	MFC 10^2^	*R*	MIC 10^2^	MFC	*R*	MIC 10^2^	MFC 10^2^
*C. albicans* ATCC 10231	1.25	2.5	2	1.25	10.0	8	2.5	10.0	4	0.625	> 10.0	> 16	1.25	2.5	2	2.5	10.0
*C. albicans* ATCC 90028	1.25	2.5	2	2.5	10.0	4	2.5	2.5	1	0.625	> 10.0	> 16	2.5	2.5	1	2.5	5.0
*C. albicans* ATCC 200955	1.25	2.5	2	2.5	10.0	4	2.5	10.0	4	1.25	> 10.0	> 8	2.5	1.0	4	2.5	5.0
*C. parapsilosis* ATCC 22019	1.25	5.0	4	2.5	5.0	2	1.25	2.5	2	1.25	> 10.0	> 8	2.5	5.0	2	0.3125	1.25
*C. krusei* ATCC 6258	2.5	5.0	2	2.5	10.0	4	2.5	2.5	1	2.5	> 10.0	> 4	2.5	5.0	2	2.5	5.0

*Note:* MIC and MFC data were expressed as µM. *R*; MFC/MIC ratio ≤ 4 means fungicidal activity and > 4 fungistatic activity.

**Table 5:** Prediction of cell line cytotoxicities of aminoantipyrine-derived Schiff bases.

Symbol	Pa values	Pi values	Cell line	Cell line full name	Tissue	Tumor type	NT. C.L.P.R
4AA-Fc	not predicted (program did not run, valence error)						
4AA-SA	0.894	0.004	HOP-62	Non-small cell lung carcinoma	Lung	Carcinoma	—
4AA-FSA	0.806	0.004	HOP-62	Non-small cell lung carcinoma	Lung	Carcinoma	—
4AA-ClFSA	0.701	0.005	HOP62	Non-small cell lung carcinoma	Lung	Carcinoma	—
4AA-FMeSA	0.730	0.005	HOP62	Non-small cell lung carcinoma	Lung	Carcinoma	—

Abbreviations: NT. C.L.P.R, Nontumor cell line prediction result; Pa, probable activity; Pi, probable inactivity.

**Table 6:** Results of ADME for pharmacokinetics, druglikeness, and medicinal chemistry of aminoantipyrine-derived Schiff bases.

	Pharmacokinetics (selected)	Druglikeness	Medicinal chemistry
(4AA-Fc)	GI absorption: High BBB permeant: Yes P-gp substrate: No CYP1A2 inhibitor: No CYP2C19 inhibitor: No CYP2C9 inhibitor: No CYP2D6 inhibitor: No CYP3A4 inhibitor: No Log Kp (skin permeation): −5.51 cm/s	Lipinski: Yes; 0 violation Ghose: Yes Veber: Yes Egan: Yes Muegge: Yes Bioavailability score: 0.55	PAINS: 0 alert Brenk: 2 alert: heavy_metal_imine_1 Leadlikeness: No; 2 violations: MW > 350, XLOGP3 > 3.5 Synthetic accessibility: 4.35

(4AA-SA)	GI absorption: High BBB permeant: Yes P-gp substrate: No CYP1A2 inhibitor: No CYP2C19 inhibitor: No CYP2C9 inhibitor: No CYP2D6 inhibitor: No CYP3A4 inhibitor: No Log Kp (skin permeation): −6.92 cm/s	Lipinski: Yes; 0 violation Ghose: Yes Veber: Yes Egan: Yes Muegge: Yes Bioavailability score: 0.55	PAINS: 0 alert Brenk: 1 alert: imine_1 Leadlikeness: Yes Synthetic accessibility: 3.05

(4AA-FSA)	GI absorption: High BBB permeant: Yes P-gp substrate: No CYP1A2 inhibitor: No CYP2C19 inhibitor: No CYP2C9 inhibitor: Yes CYP2D6 inhibitor: No CYP3A4 inhibitor: No Log Kp (skin permeation): −5.98 cm/s	Lipinski: Yes; 0 violation Ghose: Yes Veber: Yes Egan: Yes Muegge: Yes Bioavailability score: 0.55	PAINS: 0 alert Brenk: 1 alert: imine_1 Leadlikeness: Yes; Synthetic accessibility: 3.07

(4AA-ClFSA)	GI absorption: High BBB permeant: Yes P-gp substrate: No CYP1A2 inhibitor: Yes CYP2C19 inhibitor: Yes CYP2C9 inhibitor: Yes CYP2D6 inhibitor: Yes CYP3A4 inhibitor: No Log Kp (skin permeation): −5.38 cm/s	Lipinski: Yes; 0 violation Ghose: Yes Veber: Yes Egan: Yes Muegge: Yes Bioavailability score: 0.55	PAINS: 0 alert Brenk: 1 alert: imine_1 Leadlikeness: No; 1 violation: XLOGP3 > 3.5 Synthetic accessibility: 3.68

(4AA-FMeSA)	GI absorption: High BBB permeant: Yes P-gp substrate: No CYP1A2 inhibitor: Yes CYP2C19 inhibitor: Yes CYP2C9 inhibitor: Yes CYP2D6 inhibitor: Yes CYP3A4 inhibitor: No Log Kp (skin permeation): −5.44 cm/s	Lipinski: Yes; 0 violation Ghose: Yes Veber: Yes Egan: Yes Muegge: Yes Bioavailability score: 0.55	PAINS: 0 alert Brenk: 0 alert: imine_1 Leadlikeness: No; 1 violation: XLOGP3 > 3.5 Synthetic accessibility: 3.78

## Data Availability

The results of this investigation corroborate the findings and are presented within the paper.
